# High-Throughput Phenotyping of Morphological Seed and Fruit Characteristics Using X-Ray Computed Tomography

**DOI:** 10.3389/fpls.2020.601475

**Published:** 2020-11-12

**Authors:** Weizhen Liu, Chang Liu, Jingyi Jin, Dongye Li, Yongping Fu, Xiaohui Yuan

**Affiliations:** ^1^School of Computer Science and Technology, Wuhan University of Technology, Wuhan, China; ^2^Wuhan Gooalgene Technology Co., Ltd., Wuhan, China; ^3^Engineering Research Centre of Chinese Ministry of Education for Edible and Medicinal Fungi, Jilin Agricultural University, Changchun, China

**Keywords:** computed tomography, seed and fruit, morphological trait, 3D image processing, high-throughput phenotyping

## Abstract

Traditional seed and fruit phenotyping are mainly accomplished by manual measurement or extraction of morphological properties from two-dimensional images. These methods are not only in low-throughput but also unable to collect their three-dimensional (3D) characteristics and internal morphology. X-ray computed tomography (CT) scanning, which provides a convenient means of non-destructively recording the external and internal 3D structures of seeds and fruits, offers a potential to overcome these limitations. However, the current CT equipment cannot be adopted to scan seeds and fruits with high throughput. And there is no specialized software for automatic extraction of phenotypes from CT images. Here, we introduced a high-throughput image acquisition approach by mounting a specially designed seed-fruit container onto the scanning bed. The corresponding 3D image analysis software, 3DPheno-Seed&Fruit, was created for automatic segmentation and rapid quantification of eight morphological phenotypes of internal and external compartments of seeds and fruits. 3DPheno-Seed&Fruit is a graphical user interface design and user-friendly software with an excellent phenotype result visualization function. We described the software in detail and benchmarked it based upon CT image analyses in seeds of soybean, wheat, peanut, pine nut, pistachio nut and dwarf Russian almond fruit. *R*^2^ values between the extracted and manual measurements of seed length, width, thickness, and radius ranged from 0.80 to 0.96 for soybean and wheat. High correlations were found between the 2D (length, width, thickness, and radius) and 3D (volume and surface area) phenotypes for soybean. Overall, our methods provide robust and novel tools for phenotyping the morphological seed and fruit traits of various plant species, which could benefit crop breeding and functional genomics.

## Introduction

The shape and size of seeds and fruits are among the most vital agronomic traits since they play crucial parts in eating quality, yield, as well as market price. The quantitative assessment of their external and internal morphological traits could promote the progress of plant research areas including genetics, physiology, functional analysis, and plant breeding (Tanabata et al., 2012). Until now, manual measurements using the caliper and cylinder have been the most widely adopted methods. However, majority of seeds and fruits are pretty small which make the manual measurement of external morphological traits time-consuming and labor-intensive. Some shape phenotypes such as surface area and volume are three-dimensional (3D) that are almost impossible to be accurately quantified with the manual methods. Most importantly, the internal morphological traits, such as the size of air space between two cotyledons, are unable to be quantified without damaging the seeds or fruits using the manual measurement approaches. Therefore, rapid, accurate, non-destructive, and high-throughput approaches for seed and fruit phenotyping are urgently needed.

The image-based approaches using digital image processing and computer vision technologies offer solutions to automatically measure a variety of size and shape features from high-resolution images in a high-throughput way. Two-dimensional (2D) based systems are increasing used. For example, Baek et al. (2020) designed an image acquisition device that imaged 100 seeds at a time. To quantitatively measure morphological and color parameters of soybean seeds, the device was equipped with top and side-view RGB cameras. A software, SmartGrain, was developed to automatically segment individual seeds from the scanned images and measure multiple shape-related seed parameters simultaneously (Tanabata et al., 2012). Duan et al. (2011) generated a label-free facility for phenotyping of yield-related grain traits in rice, integrating the spikelet threshing, grain imaging, and real-time algorithm-based evaluation of grain traits (e.g., grain length, width, 1000-grain weight, and seed packing) for each rice plant. For fruit trait phenotyping, Ni et al. (2020) proposed a deep learning based-image processing pipeline to evaluate blueberry fruit traits including cluster compactness, fruit maturity, and berry number per cluster.

The rapid development of 3D sensing methods has led to more and more studies employing 3D imaging techniques to measure shape-related 3D traits for plants (Pieruschka and Schurr, 2019). 3D point clouds were intensively investigated for measuring surface traits at plant and canopy scales for tree and shrub crops, such as canopy volume, fruit, and flower density (Ramón et al., 2015; Underwood et al., 2016). But their resolutions are usually too low to be used for micro-dissection of seed and fruit phenotypes. The higher-resolution 3D imaging techniques such as magnetic resonance imaging (MRI) and positron emission tomography (PET) have been attempted on studying the internal structures of plant fruit, grain, and root (Jahnke et al., 2010; Borisjuk et al., 2012; Hubeau and Steppe, 2015). However, both MRI and PET scanners were not widely used in the field of plant phenotyping because of the high cost of the equipment and maintenance.

X-ray computed tomography (CT) is a non-invasive and cost-effective 3D imaging technique based on differential X-ray attenuation by object materials. Similar to MRI and PET, this technique was originally developed as a medical diagnostic tool. But it has since been applied to a broad range of fields, like material, earth, natural, and animal sciences (Cnudde et al., 2006; Schambach et al., 2010). Recent improvements in scanning quality, resolution, and speed allowed it to be adopted to visualize and quantify complex plant traits (Dhondt et al., 2010; Hughes et al., 2017; Xiong et al., 2019; Li et al., 2020; Yang et al., 2020). For example, Arendse et al. (2016) used the X-ray CT to non-destructively quantify the external and internal morphological features (volumes of aril, peel, kernel, juice content, and air space) of pomegranate fruit. Hu et al. (2020) scanned a rice spike and achieved an automatic segmentation of individual grain and high-throughput extraction of 22 spike and grain traits such as grain number, size, shape, and density. These examples demonstrated the feasibility of using CT images to measure plant traits. However, this method has not been so widely applied on quantification of general plant traits as it is expected. One of the important reasons is that the commercially available benchtop CT scanners are usually designed for medical and industrial usage rather than plant phenotyping, and therefore their sample loading carousels are not suitable for scanning plant samples with too small or too large sizes.

Here, we report the development of a low-cost and easy-to-replicate approach for imaging small seeds or fruits in batches using a commercial industrial CT scanner. The corresponding 3D image analysis software, 3DPheno-Seed&Fruit, was also developed that allows not only to extract external morphological phenotypes of seeds or fruits but also non-destructively measure their internal features. To demonstrate the practicality of our method, soybean (*Glycine max*), wheat (*Triticum aestivum*), peanut (*Arachis hypogaea*), pine nut (*Pinus koraiensis*), pistachio nut (*Pistacia vera* L.), and fruit of dwarf Russian almond (*Prunus tenella*) were examined.

## Materials and Methods

### X-Ray CT Imaging

With the aim of scanning multiple seeds and fruits at once, we mounted a plastic container with multiple sub-boxes onto the loading carousels of X-ray CT scanner. The size of the sub-boxes was adjustable that can hold the seeds and fruits with different sizes tightly and avoid shifting position during scanning. Containers were captured using the X-ray micro-CT scanning system AL-μCT-9002 (Dandong Aolong Radiative Instrument Group Co., Ltd., Dandong, Liaoning, China) with the supplied software. Focal length of CT scanner was 5 μm, the effective area is 120 mm × 120 mm, and voltage and current were set as 100 kV and 20 μA, respectively. Helical scan was conducted using the fast and continuous scan mode and its moving height could be up to 200 mm. The container was rotated in the clockwise direction. CT image reconstruction was achieved using a Feldkamp-type (FDK) come-bean CT reconstruction algorithm incorporated in the MiHitect v1.0 software (Dandong Aolong Radiative Instrument Group Co., Ltd., Dandong, Liaoning, China). The output images were saved slice by slice along the z-direction in BIN format with a final resolution of 0.1 mm (1024 × 1024).

### 3DPheno-Seed&Fruit: A CT Image Analysis Software for Extracting Seed/Fruit Morphological Phenotypes

We developed a software, 3DPheno-Seed&Fruit, for extracting the morphological features of seeds/fruits from CT image analysis with sophisticated visualization of the final results and user-interaction. It was written in Visual C++ in the Microsoft Visual Studio 2015 software creation tool and ran under Microsoft Windows 10. A graphical user interface (GUI) program was created with Qt 5.12 framework (The Qt Company Ltd.). Functions of 3D visualization and view rendering were based on the open-source Visualization Toolkit (VTK). All the CT image analysis pipelines were written in MATLAB 2014a (MathWorks, United States) and packaged into the 3DPheno-Seed&Fruit software by MATLAB Compiler^TM^. 3DPheno-Seed&Fruit software and CT image datasets used in this manuscript are free for academic purpose and can be downloaded from http://www.wutbiolab.com/resources/39/info/29 and https://github.com/whut-biolab-liuchang/project. The installation manual is available in the Supplementary File 1 and the above website.

**FIGURE 1:**
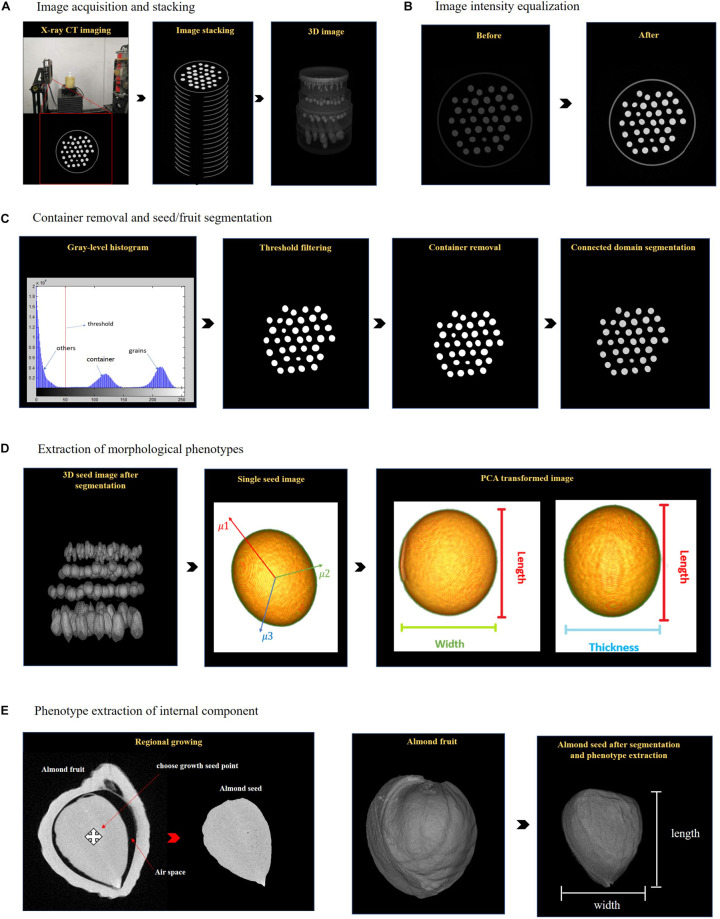
X-ray computed tomography image acquisition and processing pipeline for high-throughput phenotyping morphological characteristics of seeds/fruits. **(A)** Image acquisition and 3D reconstruction. **(B)** Image intensity equalization. **(C)** Container removal and object plant tissue segmentation. **(D)** Morphological phenotype extraction. **(E)** Internal component segmentation and phenotype extraction.

The main CT image analysis pipelines included image preprocessing, segmentation of seeds/fruits, and extraction of phenotypes, which were exhibited in Figure 1. The details are described as follows: (1) all the slices of CT image data were stacking along z-direction to form 3D images; (2) intensity standardization was conducted across samples; (3) the container for holding seeds/fruits was removed; (4) seeds/fruits were segmented out individually from the 3D images; (5) the external morphological phenotypes of seeds/fruits were measured; (6) the internal components of seeds/fruits were further separated from the whole seeds/fruits, and the corresponding morphological properties were extracted.

### Image Preprocessing

The inputs of the 3DPheno-Seed&Fruit software were all the CT slices collected from the individual sample with the format of 8-bit PNG, JPG, or TIFF. By combining them along the z-direction, the 3D images were easily gained and would be directly used for all the following image analyses. Comparing to the methods based on 2D image processing (Hughes et al., 2017), this strategy is more precise to identify seeds/fruits from the background noise and the tightly connected interlayers of container because it simultaneously took all voxels from all slices into consideration (Xiong et al., 2019). Before segmentation of seeds/fruits, we conducted a series of 3D image preprocessing, consisting of intensity standardization and removal of holder and background.

The intensity of CT images might vary across sample sets when scanning at different time and using different scanning parameters. To make sure all the images with consistent parameters, a linear stretch mapping method which was adopted in Xiong et al. (2019) was utilized to automatically standardize the intensity of CT images of all sets. The equation for linear stretch mapping is as follows:

$$x_{o\,u\,t\,\left(x,y,z\right)}=\frac{x_{i\,n\,\left(x,y,z\right)}-\text{min}\,\left(x_{i\,n}\right)}{\max\left(x_{i\,n}\right)-\text{min}\,\left(x_{i\,n}\right)}$$

where *x*_*in*(*x*,*y*,*z*)_ is the intensity of image *x* at the coordinate x, y, and z; min(*x*_*in*_) is the minimum intensity of all the input CT images; *max*⁡(*x*_*in*_) is the maximum intensity of all the input CT images; *x*_*out*_ is the output of the images after intensity standardization (Figure 1B).

### Container Removal and Seed/Fruit Segmentation

We made a seed-fruit container with multiple sub-boxes to fix seeds/fruits in position during scanning (Figure 1A). In the 3D CT image, seeds/fruits were separated by the sub-boxes, and each seed/fruit was surrounded by the edge of the sub-box. Considering edges would be removed for further image processing, we selected low-density plastic materials which have distinct attenuation characteristic of X-ray absorption from that of plant tissues (Figure 1C). The background of the 3D image is composed of noise, artifacts, and nearly black pixels (not zero intensity for most cases). Therefore, seeds/fruits have the stronger intensity compared to the container edge and background. A fixed and learned threshold of intensity has the capacity of removing majority of the container and background.

After image intensity threshold segmentation, besides the seeds/fruits, there are a small number of broken pieces of the container edges and background left in the 3D image. To completely get rid of them, we used the connected domain segmentation method and filtered out any small pieces less than one-twentieth of the mean plant tissue volume. The cleaned seed/fruit 3D image for feature extraction was obtained to generate a mask. The mask was then applied to the original 3D image to separate the 3D seed/fruit object (Figure 1C). The 3D object consisted of voxels with three coordinates and grayscale values.

### External Phenotype Extraction

After container removal and object segmentation, voxel set belonging to individual seeds/fruits was accurately obtained, which can be directly used for assessment of their external morphological features (Figure 1D). The size-related traits including length, width, thickness, and radius were measured by application of principle component analysis (PCA). We estimated the volume trait by counting the number of voxels in the point set. The surface area of seed/fruit were measured by reconstructing the seed/fruit surface using the 3D surface reconstruction algorithm. Those morphological phenotypes are important in the quality evaluation and crop breeding of seed and fruit traits.

#### Size-Related Phenotypes

The segmented seed/fruit may be tilted with irregular shape in the 3D image, which is difficult to directly use its distance on the *z*-axis to represent the length (Figure 1D). Therefore, we applied PCA to transform the object seed/fruit to another 3D space before quantify its length, width, thickness, and radius. The original 3D coordinates (x-y-z) were defined for all the voxels of each seed/fruit and used as the input for PCA. By orthogonal transformation, the voxels were converted to a set of linearly uncorrected variables (μ_1_,μ_2_,μ_3_,…,μ_*n*_) which are principle components (PCs). The three PCs with highest eigenvalues (μ_1_μ_2_μ_3_…μ_*n*_) were selected and their corresponding eigenvectors (*v*_1_,*v*_2_,*v*_3_) were the new coordinates of the voxels. By projecting the voxels toward each eigenvector, the length was calculated by measuring the size of the major axis, while the width and thickness were examined from the major and minor 2D axis of the cross section of each seed/fruit (Figure 1D). The radius is defined as the half of the mean of width and thickness (Equation: radius$=\frac{1}{2}\times \frac{\left(\text{width}+\text{thickness}\right)}{2}$).

#### Shape-Related Phenotypes

Superior to the traditional 2D images, the 3D CT images enable us to extract the shape-related characteristics of seed/fruit, including volume, surface area, compactness, and sphericity. After obtaining the voxel set of each seed/fruit, its volume is directly calculated by counting the number of voxels. However, the surface area of each 3D reconstructed seed/fruit is jagged and not smooth. Thereby, it is not accurate to simply count the outermost voxels as the exposed surface area. To smooth the surface, we reconstructed the voxel rectangle of outermost surface of seed/fruit to voxel triangle by using marching cubes 3D surface construction algorithm (Lorensen and Cline, 1987). Then we summed area of triangles to estimate the surface area.

Compactness and sphericity are two commonly used parameters that describe how closely the shape of an object resembles a perfect circle in 2D plane and sphere in 3D plane, respectively (Rosenfeld and Kak, 1976; Sakai and Yonekawa, 1992). We treated the cross section of the seed/fruit as an ellipse and its 3D shape as an ellipsoid. Compactness is defined as the ratio of the area of the sample to the area of a circle with the same perimeter. The equation is as follows:

$$C=\frac{4\,\pi \,S}{R^{2}}.$$

Where *C* is the compactness (dimensionless); *S* is the area of cross-section of the sample; and *R* is the perimeter of the sample. Circle is used as it is the most compact shape with a compactness value of 1. As the compactness approaches to 1, the cross-section of the seed/fruit approaches to a circle.

The sphericity of a sample is defined as the ratio of the surface area of an equal-volume sphere to the surface area of the sample, which extends the definition of compactness from 2D to 3D. The equation for sphericity is as follows:

$$E=\frac{S_{e}}{S_{a}}.$$

Where *E* is the sphericity (dimensionless); *S*_*e*_ is the surface area of an equal-volume sphere; *S*_*a*_ is surface area of the sample. The sphericity of a perfect spherical object is 1. As the sphericity value approaches to 1, the seed/fruit sample approaches to a sphere.

### Segmentation of Internal Components and Phenotype Extraction

Besides quantification of the external morphological phenotypes, CT images enable to non-destructively quantify the morphological features of internal components (such as arils, peel, kernel, and air space) of seeds/fruits (Figure 1E). Before phenotype extraction, we need to segment the voxel set belonging to each component from the entire voxel set of the seed/fruit. Since the voxels of the same internal component and adjacent to each other have relatively homogeneous grayscale properties, we applied a 3D region growing algorithm (Sekiguchi et al., 1994) for segmentation. The procedures are described as follows: (1) the centroid of each seed/fruit internal components was determined as the 3D region growing seed point; (2) the region growing started from the seed voxel and grow recursively if its 6-adjacent neighborhood satisfied the growing criteria; (3) the region stopped growing when no new seed is found. The growing criteria is that a pixel *P*_*x*_ is merged into the growing region if its intensity *i*_*P_x*_ is smaller than the averaged intensity of the growing region $\sum_{j}^{x-1}i_{P_{j}}$. The idea is expressed by the following equation: $\left|i_{P_{x}}-\frac{\sum_{j}^{x-1}i_{P_{j}}}{x-1}\right|\leq \alpha$ where α is a user-specified threshold. We set α to 10 in this study. After segmentation of each internal component, the size and shape-related phenotypes were extracted by using the same pipeline as the external phenotypes of seeds/fruits.

### Manual Measurement of Phenotypes

The morphological phenotypes (length, width, thickness, and radius) of soybean and wheat seeds were manually measured by using a micrometer caliper. Each measurement was repeated three times and the average value was used as the reference data.

### Statistical Analysis

All statistical analyses were carried out with R version 3.6 (R Foundation for Statistical Computing, Vienna, Austria). Pairwise correlation analysis among eight morphological traits of soybean seeds extracted by 3DPheno-Seed&Fruit software were computed using Pearson correlation coefficients. To compare the difference between the 3DPheno-Seed&Fruit and manual measurements for soybean and wheat seeds, we fitted the data to a simple linear regression using the CT measurement as *y*-axis and the manual measurement as *x*-axis. The coefficient of determination (*R*^2^), root mean square error (RMSE), and mean absolute percentage error (MAPE) were calculated with the following equations:

$$R^{2}=1-\frac{\sum_{i}{\left(x_{i}-y_{i}\right)}^{2}}{\sum_{i}{\left(x_{i}-\bar{y}\right)}^{2}}$$

$$R\,M\,S\,E=\sqrt{\frac{\sum_{i}{\left(x_{i}-y_{i}\right)}^{2}}{n}}$$

$$M\,A\,P\,E=\frac{1}{n}\,\sum_{i}\frac{\left|x_{i}-y_{i}\right|}{x_{i}}\times 100\%$$

Where n is the total number of measurements; *x*_*i*_ is the manual measurement results; *y*_*i*_ is the CT measurement results, and $\bar{y}$ is the mean of the CT measurements.

## Results and Discussion

### 3DPheno-Seed&Fruit Software Description

3DPheno-Seed&Fruit is a high-throughput CT image analysis software that can be used for extracting eight morphological traits of seeds/fruits (length, width, thickness, radius, surface area, volume, compactness, and sphericity) from 3D images. It analyses X-ray CT images that are stored in a specific folder. For all the inputs of CT slices, 3DPheno-Seed&Fruit software automatically stacks them along z-direction to form the 3D image and equalizes the image intensity across all the inputs to minimize image noises. The user-defined intensity threshold parameter is set for removing the container that holds seeds and fruits during scanning. Then the software can automatically segment out the seeds/fruits and measure their external morphological phenotypes individually using the “ExternalPheno” plugin. For extraction of morphological features of inner components, such as extraction of the seed volume inside the draft Russian almond fruit, the region growing function serves for segmentation of internal components and then extract their eight morphological features. The segmentation results which are the CT images of individual seeds/fruits are automatically exported in the PNG (portable network graphics) format, together with the phenotype results being exported as a CSV (comma-separated values) file.

**FIGURE 2:**
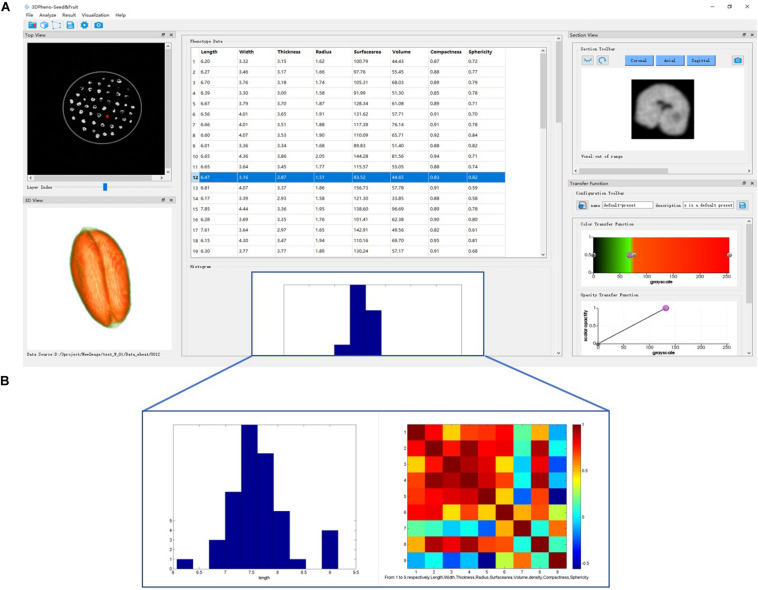
3DPheno-Seed&Fruit software interface with wheat data. **(A)** The main interface. **(B)** Example of histogram and correlation plots incorporated in the lower-middle of the interface.

The outstanding advantages of the 3DPheno-Seed&Fruit software is the sophisticated visualization of the phenotype results and user-interaction. As shown in the interface (Figure 2), the end-users are allowed to visualize the original CT images (with a batch of seeds and container) after stacking in a top-view (in the top-left of the interface), a spreadsheet of morphological phenotype results (in the top-middle of the interface), and the statistical graphs (histograms and correlations) summarizing the phenotype results (in lower-middle of the interface). In the result spreadsheet, phenotypes extracted from individual seeds/fruits were saved by row. Through double-clicking a row of the spreadsheet, the corresponding seed will be highlighted in the top-view original CT image window. In the same time, the image of selected seed/fruit after segmentation in the cross-sectional and 3D view will be exhibited in the top-right and the lower-left of the interface, respectively. The color and transparency of the selected single seed/fruit in the 3D view window can be adjustable according to the purpose of end-user, and these functions can be found in the lower-right of the interface.

### Applications of Seed/Fruit Container and 3DPheno-Seed&Fruit Software

We attempted our proposed imaging strategy and the 3DPheno-Seed&Fruit software to extract morphological features of seeds/fruits from the CT images. Our imaging strategy is to mount the special-designed seed/fruit container onto the loading carousels of the X-ray CT scanner (Figure 1A). Since the effective area of the X-ray CT scanner AL-μCT-9002 is 120 mm × 20 mm, we designed a series of cylindrical-shaped containers with various diameters of 70, 80, and 90 mm, respectively. The height of these cylindrical containers was designed to be consistent as 40 mm. Since the maximum perpendicular distance that the loading carousels of X-ray CT scanner is 200 mm during the helical scanning, up to five containers can be scanned at one time. For the convenience of container removal in the image processing step, we chose the low-density plastic material to make the container, whose image intensity is far less than our plant tissues (Figure 1C, gray-level histogram). To hold seeds/fruits in batches and fix each seed/fruit in position, the containers were subdivided into multiple sub-boxes using the soft polyurethane foam, which allows to adjust the size of the sub-box according to the seed size. Therefore, the number of seeds that each container can hold mainly depends on the types and sizes of seeds.

**FIGURE 3:**
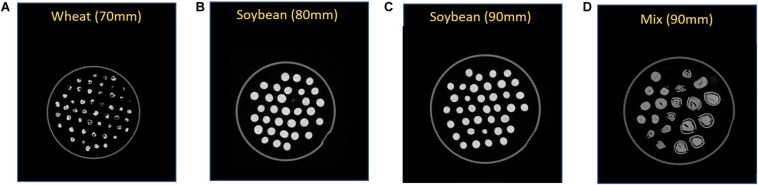
The raw X-ray computed tomography images. **(A)** wheat seeds in the container with a diameter of 70 mm; **(B)** soybean seeds in the container with a diameter of 80 mm; **(C)** soybean seeds in the container with a diameter of 90 mm; **(D)** peanuts, pine nuts, and pistachio nuts in the container with a diameter of 90 mm.

As an example, we tested the proposed imaging strategy by scanning a pile of four containers together, as shown in Figure 1A. Among them, one container with a diameter of 70 mm was filled with 50 wheat grains, two containers with diameters of 80 and 90 mm were filled with 30 and 36 soybean seeds, respectively, and one container with a diameter of 90 mm was filled with a combination of eight peanuts, seven pine nuts, and six pistachio nuts (Figure 3). The total scanning time for four containers was 5 min, and reconstruction time was 7 min. In the end, we obtained a total of 1,600 CT slices for four containers with only half of them containing seeds and the other slices only containing edges of containers and sub-boxes (Table 1). According to this finding, for the small seeds like soybean and wheat, we can reduce the height of the cylindrical container and piled more containers together for scanning to improve the scanning throughput of seeds.

**TABLE 1:** Summary of total CT slides, effective CT slides, and image analysis times for different sizes of containers.

**Container diameter (mm)**	**Total No. of CT slides**	**Effective No. of CT slides**	**Image analysis time (min)**
70 (Wheat)	400	129	1.5
80 (Soybean)	400	130	1.5
90 (Soybean)	400	140	1.5
90 (Mix)	400	273	2.5

3DPheno-Seed&Fruit software was applied to automatically extract the eight seed phenotypes (length, width, thickness, radius, surface area, volume, compactness, and sphericity) in a fast speed of a few minutes (Table 1). An example of phenotype extraction using 3DPheno-Seed&Fruit software was shown in Supplementary Video 1. Considering that analyzation of the 1,600 CT slices collected from four containers together would consume too much memory which caused software crashed in personal computer, CT slices belonging to each container were inputted together into the software. The average time for extraction of eight seed phenotypes costed about 1.5∼2.5 min per container (Table 1).

Seed phenotype results of wheat, soybean, peanut, pine nut, and pistachio nut were summarized in Table 2. As expected, soybean and pistachio nut had had lower correlation of variation (cv) values of majority seed traits than wheat, peanut, and pine nut, because more consistency of size and shape were observed for soybean and pine nut seeds than the other crop seeds in the CT images (Figure 3). The consistency between observation and measurement gives confidence to our proposed CT methods.

**TABLE 2:** The summary of external morphological phenotypes of soybean, wheat, peanut, pine nut, and pistachio nut seeds extracted from X-ray CT images using the 3DPheno-Seed&Fruit software.

**Morphology trait**	**Soybean (66)**		**Wheat (50)**		**Peanut (8)**		**Pine nut (7)**		**Pistachio nut (6)**	
	**Range**	**cv**	**Range**	**cv**	**Range**	**cv**	**Range**	**cv**	**Range**	**cv**
Length (mm)	5.96–7.61	0.054	5.20–7.85	0.076	14.40–18.45	0.083	13.75–16.66	0.075	18.70–21.77	0.081
Width (mm)	5.71–7.12	0.053	3.16–4.56	0.090	7.92–10.02	0.15	8.93–11.49	0.10	12.81–13.95	0.046
Thickness (mm)	4.97–6.98	0.070	2.88–3.86	0.079	6.96–10.02	0.14	6.22–9.17	0.17	11.61–11.99	0.018
Radius (mm)	2.72–3.53	0.057	1.51–2.05	0.075	3.78–5.49	0.14	3.87–5.23	0.11	6.17–6.39	0.019
Surface area (mm^2^)	117.60–190.00	0.11	56.08–127.26	0.19	352.87–602.18	0.19	385.28–890.02	0.30	1151–1742.6	0.062
Volume (mm^3^)	91.74–181.09	0.16	21.30–60.84	0.22	402.04–859.07	0.28	364.38–729.73	0.28	1153–1325.3	0.071
Compactness	0.99–1.00	0.0018	0.82–0.96	0.031	0.81–0.97	0.059	0.89–0.95	0.035	0.91–0.95	0.024
Sphericity	0.81–0.90	0.022	0.50–0.86	0.114	0.79–0.88	0.038	0.53–0.73	0.12	0.41–0.45	0.042

*cv denotes the correlation of variation across multiple seeds of each crop species. Seed numbers of each crop species were present in the bracket.*

The 3DPheno-Seed&Fruit software was also applied to quantify the fruit morphological phenotypes of dwarf Russian almond. Besides extracting the external profile features, we used the “InternalPheno” plugin incorporated in 3DPheno-Seed&Fruit to extract the almond seed phenotypes inside the fruit. Eight morphological phenotypes measured for both external almond fruit and internal almond seed were listed in Table 3, and the measurement process was shown in Figure 1E. Therefore, our software is versatile for evaluating the external structure of seeds and fruits and their internal compartments non-invasively.

**TABLE 3:** The fruit and seed phenotypes of draft Russian almond extracted from X-ray computed tomography images using the 3DPheno-Seed&Fruit software.

**Morphological trait**	**Fruit (External)**	**Seed (Internal)**
Length (mm)	59.85	32.7
Width (mm)	43.11	24.86
Thickness (mm)	34.18	19.82
Radius (mm)	19.32	11.17
surface area (mm^2^)	8837.25	2129.75
Volume (mm^3^)	19503	6504.64
compactness	0.96	0.97
Sphericity	0.58	0.96

*Air space volume means the volume of air space between almond fruit and seed.*

### Comparisons Between CT and Manual Measurements

**FIGURE 4:**
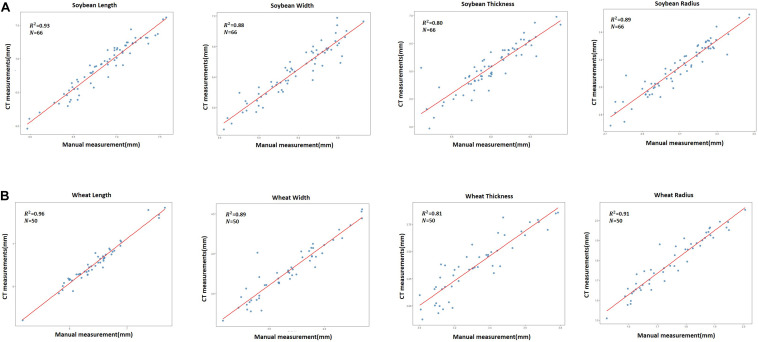
Comparison between the 3DPheno-Seed&Fruit and manually measured phenotypes of seeds using the simple linear regression. **(A)** soybean; **(B)** wheat. Each point represents individual seeds. Coefficient of determination (*R*^2^) and number of observations (*N*) are shown in individual scatter plots.

To verify the accuracy of phenotypes extracted by our CT image methods, we compared their phenotype results with these measured by hand. Seed length, width, thickness, and radius were measured by hand for 66 soybean seeds and 50 wheat seeds whose phenotypes had been measured by the 3DPheno-Seed&Fruit software previously. Regression analysis were conducted, and results are present in Figure 4. These size-related traits were highly consistent between the manual and CT methods for both soybean and wheat seeds, even though wheat seeds had a slightly larger *R*^2^, smaller MAPE, and smaller RMSE than soybean seeds (Table 4). The high consistencies confirmed the reliability of our CT measurements.

**TABLE 4:** Comparison analysis results between 3DPheno-Seed&Fruit and manual measurements for soybean and wheat seed phenotypes.

	**Soybean**			**Wheat**		
Morphological trait	***R*^2^**	**MAPE (%)**	**RMSE**	***R*^2^**	**MAPE (%)**	**RMSE**
Length	0.93	1.40	0.11	0.96	1.74	0.092
Width	0.88	2.05	0.12	0.89	3.30	0.11
Thickness	0.80	2.43	0.18	0.81	3.20	0.092
Radius	0.89	1.89	0.057	0.91	2.62	0.035

*R^2^ denotes the coefficient of determination for linear regression; RMSE stands for root mean square error; MAPE stands for mean absolute percentage error.*

### Pairwise Correlations Among CT Measured Traits

**FIGURE 5:**
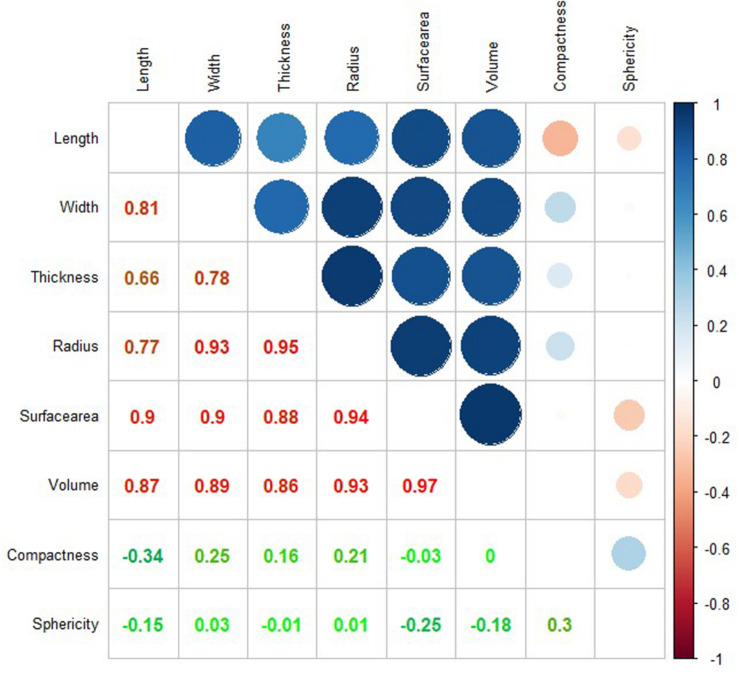
Pairwise-correlation coefficients among eight morphological phenotypes of soybean seeds.

We explored the correlations between each pair of eight phenotypes that extracted by the CT methods from 66 soybean seeds. The correlation coefficients among all the eight phenotypes were shown in Figure 5. Several expected or intuitive correlations were detected, such as seed volume being strongly correlated with seed surface area (correlation coefficient *R* = 0.97). In addition, seed volume had higher correlations with seed width (*R* = 0.89), seed radius (*R* = 0.93) than seed thickness (*R* = 0.86), and seed length (*R* = 0.87). These results indicate that seed width and radius have greater effects on seed volume than seed thickness and length. The similar trends were also observed in rice (Hu et al., 2020). Seed compactness and sphericity showed lower correlations with other size and shape-related features. Moreover, negligible correlations were observed between seed sphericity and seed width (*R* = 0.03), radius (*R* = 0.01), thickness (*R* = −0.01), length (*R* = −0.15), and volume (*R* = −0.18), indicating that the seed width, radius, thickness, length, and volume were not the main effects on the extent of a seed resembling sphere in 3D plane.

## Conclusion

In this study, we propose a high-throughput method of precisely investigating morphological phenotypes on seeds and fruits using X-ray CT scanning technology. The specially designed seed-fruit container enables to scan seeds and fruits in batches, and the corresponding 3D image analysis software, 3DPheno-Seed&Fruit, can automatically segment individual seeds/fruits and extract eight morphological traits of their internal and external compartments in a fast speed. Using the proposed methods, we successfully characterized morphological features of soybean, peanut, wheat, pine nut and pistachio nut seeds. For draft Russian almond, the external features of the entire fruit and seed phenotypes inside the fruit endocarp were measured by using the 3DPheno-Seed&Fruit software. Compared to 2D imaging methods (Tanabata et al., 2012; Evgenii et al., 2017; Baek et al., 2020), our methods quantify 3D morphological traits, such as the surface area, volume, and sphericity. Compared to the other 3D image analysis pipelines designed for grain phenotyping (Glidewell, 2006; Hughes et al., 2017; Xiong et al., 2019; Hu et al., 2020; Li et al., 2020), our methods provide an additional function of non-destructively measuring morphological phenotypes of seed and fruit internal compartments. The GUI design software, 3DPheno-Seed&Fruit, is quite user-friendly, which is easy to navigate and has the excellent visualization functions for displaying phenotyping results. In one word, our methods are powerful tools in seed and fruit 3D phenotyping, which will enhance the efficacy and accuracy of seed and fruit evaluation and eventually benefit the seed and fruit industries and crop breeding society.

## Data Availability Statement

The datasets presented in this study can be found in online repositories. The names of the repository/repositories and accession number(s) can be found in the article/Supplementary Material.

## Author Contributions

WL, XY, and YF designed and managed the study. WL wrote the manuscript. CL and WL conducted the data analyses and interpreted the results. CL and DL collected CT images. WL, CL, JJ, DL, YF, and XY developed the CT image analysis software. All authors edited the manuscript and approved the final version.

## Conflict of Interest

JJ and DL were employed by the company Wuhan Gooalgene Technology Co., Ltd. The remaining authors declare that the research was conducted in the absence of any commercial or financial relationships that could be construed as a potential conflict of interest.

## Supplementary Material

Supplementary Video 1 3DPheno-Seed&Fruit software introduction and a demo for extracting soybean seed phenotypes from X-ray computed tomography images.

Supplementary File 1 3DPheno-Seed&Fruit software installation manual.
