# Supplementary material for: High-Throughput Phenotyping of Morphological Seed and Fruit Characteristics Using X-Ray Computed Tomography
Source: Front Plant Sci. 2020 Nov 12;11:601475. doi: 10.3389/fpls.2020.601475 (PMC7688911; doi:10.3389/fpls.2020.601475)
Supplement: Supplementary File 1 — 3DPheno-Seed&Fruit software installation manual. [file Data_Sheet_1.DOCX]

**High-throughput phenotyping** **of morphological seed and fruit characteristics using** **X-ray** **computed tomography**

Weizhen Liu^1^, Chang Liu^1^, Jingyi Jin^2^, Dongye Li^2^, Yongping Fu^3*^, and Xiaohui Yuan^1^^,3*^

^1^School of Computer Science and Technology, Wuhan University of Technology, Wuhan, Hubei 430070, China

^2^Wuhan Gooalgene Technology Co., Ltd, Wuhan, Hubei 430070, China

^3^Engineering Research Centre of Chinese Ministry of Education for Edible and Medicinal Fungi, Jilin Agricultural University, Changchun 130118, China

*Correspondences: Xiaohui Yuan, email: [yuanxiaohui@whut.edu.cn](mailto:yuanxiaohui@whut.edu.cn); Yongping Fu, email: [fuyongping@jlau.edu.cn](mailto:fuyongping@jlau.edu.cn)

**Supplemental File 1: 3DPheno-Seed&Fruit Software Installation Manual**

**1. INTRODUCTION**

The software is designed for bulk extraction of seeds or fruit phenotypes from X-ray CT images. It is created based on the open-source visualization toolkit (VTK), programming software Microsoft Visual Studio 2015 and Matlab2014a. Our software needs to run under the Microsoft windows10 system.

**2. SOFTWARE DOWNLOAD**

The software is free for academic purpose and available at <http://www.wutbiolab.com/resources/39/info/29> or https://github.com/whut-biolab-liuchang/project.

**3. SOFTWARE INSTALLATION**

Click the zip file
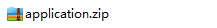
to install the software to your computer. After unzipping the file, please open the
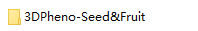
 folder that contains our software (3DPheno-Seed&Fruit.exe) and various environment configuration files.


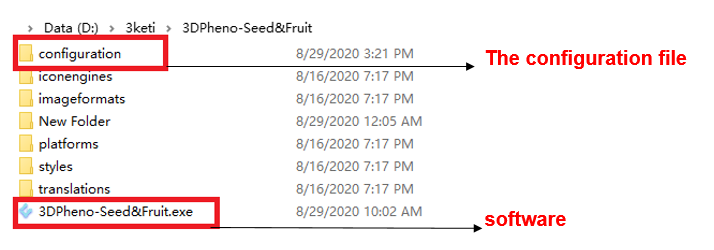


**3.1 System Environment Configuration**

Firstly, please open the **configuration folder**, and then click
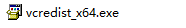
 to install the Microsoft Visual C++ 2005 runtime libraries (If your computer has never been installed with Microsoft Visual C++ 2005 runtime libraries before, you will need to restart your computer after configuring it. If you have installed this runtime before, this step can be skipped.)

**
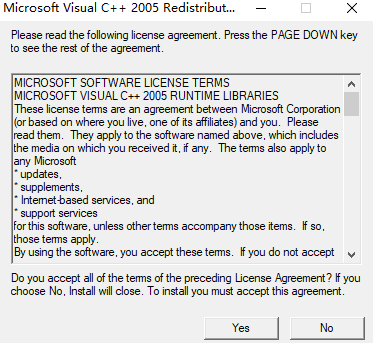
**

**3.2 Installation Options**

Click **myappinstaller_mcR.exe** to install the plug-ins needed to run the software and the configuration environment required for the software (You need to connect to the network and close the network firewall).

(1) Select installation location.


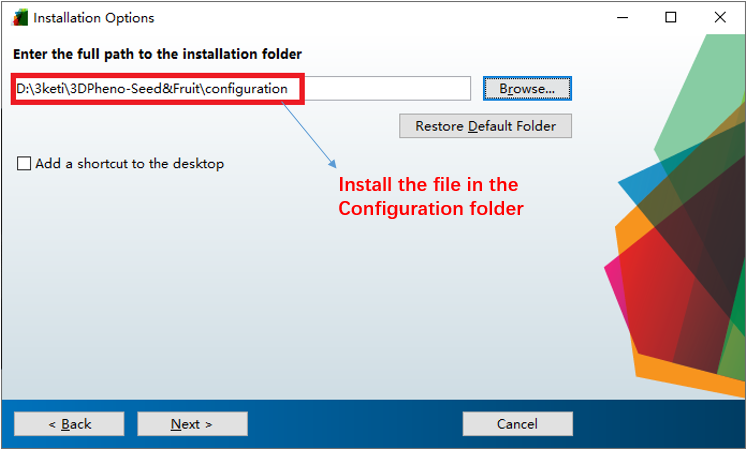


(2) Install the MATLAB environment.


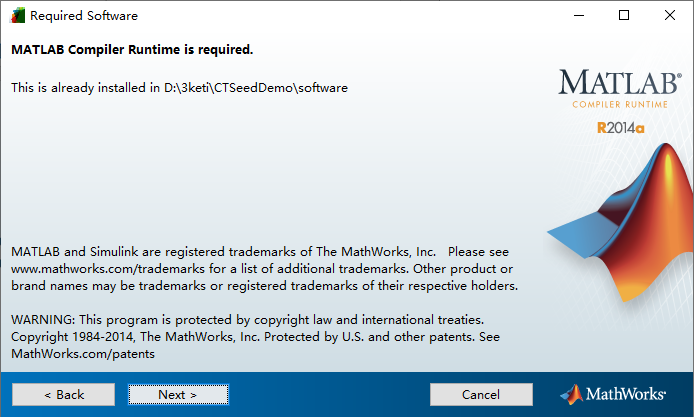


(3) Confirm the installation


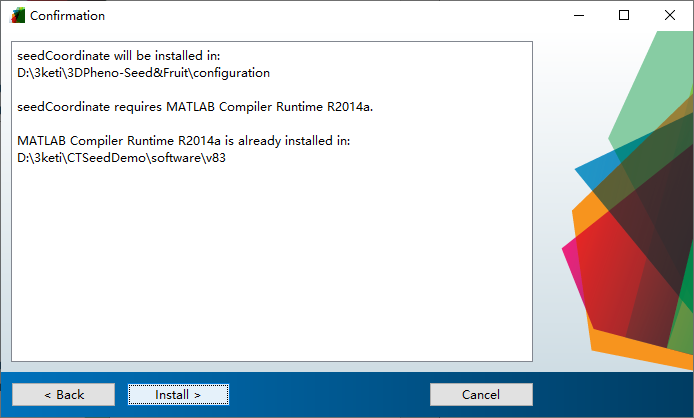


**3.3 File and Shortcut/Icon Locations**

If you do not choose a custom installation, the plug-in and plug-in configuration files will by default be placed in a file named C:\Program Files\. The executable software is in the 3Dpheno-Seed&Fruit folder, which has the same name as the folder. You can use it when the installation is complete. Please watch the video provided by us before using it. The video introduces the operation procedures and functions in the video.
